# Supplementary material for: Identification and functional analysis of non-coding regulatory small RNA FenSr3 in Bacillus amyloliquefaciens LPB-18
Source: PeerJ. 2023 May 15;11:e15236. doi: 10.7717/peerj.15236 (PMC10194069; doi:10.7717/peerj.15236)
Supplement: Supplemental Information 4 [file peerj-11-15236-s004.zip › KO/CK-vs-T1_map/map04626.html]

KEGG PATHWAY: Plant-pathogen interaction - Reference pathway


|  |  |
| --- | --- |
| **Plant-pathogen interaction - Reference pathway** |  |

[
Pathway menu
| Organism menu
| Pathway entry
| Show description
| User data mapping
]

Plants lack animal-like adaptive immunity mechanisms, and therefore have evolved a specific system with multiple layers against invading pathogens. The primary response includes the perception of pathogens by cell-surface pattern-recognition receptors (PRRs) and is referred to as PAMP-triggered immunity (PTI). Activation of FLS2 and EFR triggers MAPK signaling pathway that activates defense genes for antimictobial compounds. The increase in the cytosolic Ca2+ concentration is also a regulator for production of reactive oxygen species and localized programmed cell death/hypersensitive response. The secondary response is called effector-triggered immunity (ETI). Pathogens can acquire the ability to suppress PTI by directly injecting effector proteins into the plant cell through secretion systems. In addition, pathogens can manipulate plant hormone signaling pathways to evade host immune responses using coronatine toxin. Some plants possess specific intracellular surveillance proteins (R proteins) to monitor the presence of pathogen virulence proteins. This ETI occurs with localized programmed cell death to arrest pathogen growth, resulting in cultivar-specific disease resistance.

|  |  |  |
| --- | --- | --- |
| Reference pathway | 184% 150% 122% 100% 82% 67% 55% | 图片下载 |
